# Analysis of Specific Perfluorohexane Sulfonate Isomers by Liquid Chromatography-Tandem Mass Spectrometry: Method Development and Application in Source Apportionment

**DOI:** 10.1155/2022/8704754

**Published:** 2022-09-22

**Authors:** Liping Yang, Xin Chen, Lingyan Zhu, Yixin Wang, Guoqiang Shan

**Affiliations:** Key Laboratory of Pollution Processes and Environmental Criteria, Ministry of Education, Tianjin Key Laboratory of Environmental Remediation and Pollution Control, College of Environmental Science and Engineering, Nankai University, Tianjin 300350, China

## Abstract

Characterization of perfluorohexane sulfonate (PFHxS) isomers, a chemical proposed for listing under the Stockholm Convention, is important to elucidate its environmental behaviors and sources. Optimized chromatographic separation coupled with monitoring of the characteristic fragments enabled the identification of four mono-substituted and two di-substituted branched PFHxS isomers. The transitions of molecular ions *m/z* 399 to the fragments *m/z* 80 (*n*-), *m/z* 169 (*iso-*), *m/z* 319 (*1m-*), *m/z* 80 (*2m*-), and *m/z* 180 (*3m*-) were selected for quantifying the mono-substituted isomers. Method accuracy of the established LC-MS/MS was verified by comparing the results of technical products with those determined by ^19^F-nuclear magnetic resonance (NMR). The developed method was then used to quantify the isomeric compositions of PFHxS in the perfluorooctane sulfonate (PFOS) industrial products which contained PFHxS as an impurity, as well as in several kinds of water samples, with the limits of detection for all isomers in the range of 4 to 30 pg/L. For the first time, a liquid chromatography-tandem mass spectrometry method was established to separate and quantify the PFHxS isomers. The isomeric profiling of water samples suggested that PFHxS in the waters was mainly due to the direct contamination of PFHxS rather than from PFOS contamination.

## 1. Introduction

Perfluoroalkyl acids (PFAAs), mainly including perfluoroalkyl carboxylic acids (PFCAs, C_*n*_F_2*n*+1_COOH, *n* = 4–14) and perfluoroalkyl sulfonate acids (PFSAs, C_*n*_F_2*n*+1_SO_3_H, *n* = 4, 6, 8), have been and are still being used in some chemical processing and related commercial production, due to their excellent thermal stability, chemical stability, and surface activity [1]. Because of their extensive usage and release, they are ubiquitous in the environment. Among them, perfluorooctane sulfonate (PFOS) has been documented with highly persistent, bioaccumulative, and toxic features from the environmental and human health perspectives [2], and its related chemicals were included in Annex B of the Stockholm Convention in 2009 [3]. With the global phase-out and regulations on PFOS, short-chain homologs such as perfluorohexane sulfonate (PFHxS), are being used as one typical substituent of PFOS [4]. The total emission of PFHxS from direct discharge as well as indirect source of its massive precursors was up to 1022 metric tons during 1958–2015 [5]. In addition, PFHxS often appears in PFOS industrial products as a major impurity with a content of 10–17% [6]. Consequently, PFHxS has found its occurrence in the worldwide environment even in remote polar regions [4]. A recent survey reported that PFOS, PFOA, as well as PFHxS were the prominent homologs in the surface waters of 22 developing countries [7]. Since Olsen et al. observed that the half-live of PFHxS in humans was 7.3–8.5 years, approximately 1.5 times longer than PFOS [8], many studies revealed that PFHxS also exhibited characteristics of persistent organic pollutants and displayed a variety of adverse effects [9]. In 2017–2019, PFHxS and the related compounds were proposed for listing in Annexes of the Stockholm Convention [9, 10].

Like PFOS, PFHxS and its precursors are manufactured from perfluorooctane sulfonyl fluoride synthesized by electrochemical fluorination (ECF) as a mixture of linear (*n*-) and branched (*br*-) isomers [11]. *br-*PFHxSK (potassium perfluorohexane sulfate solution/mixture of linear and branched PFHxS isomers) purchased from Wellinton lab Inc. (Canada) as an example, it is composed of 81.1% *n*-, 8.9% *iso-* (4*m*-), 2.9% 1*m*-, 1.4% 2*m*-, 5.0% 3*m*-, 0.2% 3,3*m-*, and 0.5% other unidentified isomers, measured by ^19^F-nuclear magnetic resonance (^19^F-NMR). The nomenclature for the main PFHxS isomers is provided in the Supporting Information (SI, Table S1). Many studies reported that there are different toxic effects [12] and environmental behaviors, such as partitioning and bioaccumulation among isomers of PFOS and PFHxS, which inevitably lead to their specific isomer patterns in various environmental and biological matrices [11–13]. Some studies showed that the isomer-selective behaviors of PFHxS isomers displayed differently from those of PFOS isomers [14]. Zhong et al. revealed different elimination pathways of PFHxS isomers from those of PFOS isomers [15]. Chen et al. reported that *n*-PFHxS crossed the placenta more easily than *br*-PFHxS, while the opposite trend was observed for PFOS isomers [15]. Hence, the isomer compositions can be important fingerprints to track their sources of PFAAs in the environment considering their isomer-selective environment behaviors [16, 17].

Accurate analysis of the specific isomers of PFOS or PFHxS in environmental samples is the prerequisite for isomer profiling. Although there is a solid analytical method for PFOS isomers in complex environmental samples [13], the analytical method for distinguishing branched PFHxS isomers is far from mature. Albeit ^19^F-NMR has great potential to quantify the isomers of PFOS and PFHxS in technical products, it is not suitable for complex matrices, such as environmental and biota samples, because of the high detection limits and a large amount of signal-interfering impurities [18]. Comparatively, chromatography techniques coupled with mass spectrometry (MS) provide effective separation and highly specific MS/MS transitions for analysis of trace levels of PFAA isomers, such as PFOS in complex samples [19]. Recently, Harada et al. developed an in-port derivation-gas chromatography negative chemical ionization-mass spectrometry to fully separate PFAAs including all PFHxS isomers [20]. Some attempts have been tried to develop a liquid chromatography-tandem mass spectrometry (LC-MS/MS) method to analyze PFHxS isomers because no derivation is necessary [21]. However, the individual branched isomers were neither well separated, nor well identified due to lack of commercial standards for individual PFHxS isomers [22–25]. Thus, further attempts should be enforced to make full resolution of PFHxS isomers in LC-MS/MS using the currently available mixed standards of *br-*PFHxSK. Moreover, considering the structural similarity between PFOS and PFHxS, the information on highly selective fragment ion monitoring and specific collision-induced dissociation patterns of PFOS isomers provides hints for isomer identification of PFHxS [26].

Herein, this study was designed to realize full resolution of the main isomers of PFHxS by optimizing the LC separation and characterizing specific MS transitions of individual branched isomers of PFHxS. With the help of ^19^F-NMR, specific transition ions were assigned to distinguish and quantify individual branched isomers of PFHxS in several technical products. Based on these, a reliable LC-MS/MS method was established to quantify PFHxS isomers in complex matrices such as water samples. The isomeric patterns in the environmental water samplers were compared with those in the technical products to figure out the possible source of PFHxS in the environment.

## 2. Experimental Section

### 2.1. Materials and Reagents

Three technical products of perfluorohexane sulfonate (PFHxS) (TP-1, 2, and 3) and two PFOS industrial products were obtained from domestic manufacturers in China, and the detailed information is listed in Table S2. The standard of mixed branched isomers of PFHxSK (*br*-PFHxSK) and ^18^O_2_-*n*-PFHxS, which were used as the standard and internal standard, respectively, for quantification, were purchased from Wellington Laboratories (Guelph, ON, Canada). HPLC-grade methanol and acetonitrile were acquired from Fisher Scientific Co. (Ottawa, Canada). HPLC-grade ammonium formate was obtained from Anpel lab technologies Inc. (Shanghai, China). Deionized water was used during the whole experiment.

### 2.2. Experimental Design

*Br*-PFHxSK was selected as the standard for the analysis of PFHxS isomers because the supplier provided its isomer compositions determined by ^19^F-NMR. To establish the LC-MS/MS method, chromatographic separation was firstly optimized with two sets of organic mobile phases consisting of methanol or acetonitrile. Then, the total ion chromatograph (TIC) of PFHxS was obtained by scanning the product ions of its parent ion *m/z* 399 in the range of *m/z* 60–399. Therefore, multi-reaction monitoring (MRM) ion chromatograms of the fragments of PFHxS isomers were extracted, and the characteristics product ions with the same retention times were classified as one group. By referring to the mass spectrometry fragmentation modes of PFOS isomers, the branched-chain positions of the specific PFHxS isomers were identified. Accordingly, for the first time, ^18^O_2_-*n*-PFHxS was used as the internal standard, and *Br*-PFHxSK as the mixed standard, and a quantitative LC-MS/MS method was developed to analyze PFHxS isomers. This method was applied to measure the isomeric compositions of the three PFHxS technical products, TP-1, -2, and -3. The measured results were then compared with those determined by ^19^F-NMR, to verify the accuracy of the LC/MS method. It was finally applied to measure the isomers of PFHxS in the PFOS industrial products which contained impurity of PFHxS, as well as in real environmental water samples.

### 2.3. LC-MS/MS Conditions

Isomer identification and quantification were accomplished on an LC-MS/MS operated under negative mode, using an Acquity UPLC system coupled to a Xevo TQ-S triple-quadrupole mass spectrometer (Waters, Milford, MA, USA). About 10 *µ*L of sample solution (10 mg/L in water solution for standard) was injected into a monomerically bonded perfluorooctyl Fluoro Sep-RP Octyl (PFO) column (3 *µ*m, 100 Å, 150 mm × 2.1 mm, ES Industries, USA). Two sets of mobile phases consisting of the same 20 mM ammonium formate aqueous solution as solvent A (adjusted to pH 4.0) and methanol or acetonitrile as solvent B were utilized. The flow rate was 0.25 mL/min and the elution gradient conditions are listed in Table 1. The MS/MS was operated in product ion scan mode at the collision voltage of 15, 25, 35, 45, and 55 eV, respectively. For quantitative analysis in water samples, multiple reaction monitoring (MRM) mode was used with the transition ions and optimal MS conditions listed in Table S3. A series of standard solutions (1–200 *µ*g/L) with 10 *µ*g/L ^18^O_2_-*n*-PFHxS were injected to obtain the calibration curves.

### 2.4. ^19^F-NMR Analysis

PFHxS technical product (ca. 200 mg) was dissolved in methanol-d_4_ (1.0 mL), and 10 *µ*L of hexafluorobenzene was added and then transferred into an NMR tube. A Bruker Avance DPX 400 NMR spectrometer equipped with a Bruker SEF ^19^ F/^1^H dual probe head was operated at 375.5 MHz. Assignment of the linear and branched isomers was performed according to the previous method for PFOS isomers [27–29]. For details of NMR signal assignment, see SI.

### 2.5. Water Sample Analysis

Pure water was spiked with the technical products to get a series of solutions with different concentrations, which were used to characterize the method's linearity, sensitivity, and repeatability. Seven natural water samples including three surface water samples from Haihe river (W1, W2, and W3), two surface water samples from Taihu lake, and two drinking water samples from Tianjin, China, were analyzed by the established method. About 500 mL of water sample were pretreated according to the method reported in the previous studies [30, 31], and finally were concentrated to 200 *μ*L volume. For LC-MS/MS analysis, 10 *μ*L of the extracted solution for each sample was injected into the column.

## 3. Results and Discussion

### 3.1. Chromatographic and MS/MS Resolution of PFHxS Isomers

**Figure 1:**
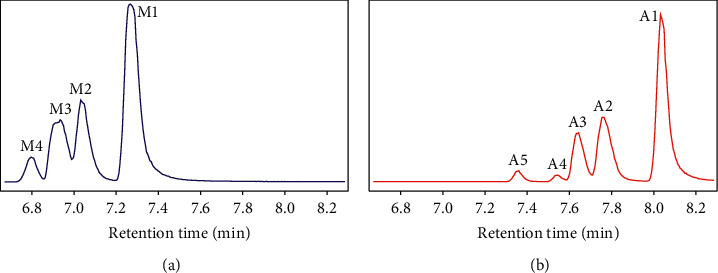
Total ion chromatograms of an ECF PFHxS technical product showing the impacts of organic reagents in the mobile phase on isomeric separation: (a): ammonium acetate (pH 4.0): methanol; (b): ammonium acetate (pH 4.0): acetonitrile.

Our previous study revealed that the resolution of PFAA isomers was distinctly affected by the organic solvent used in the mobile phase and specific monitoring daughter ions [32]. In previous studies where mobile phase consisted of water and methanol, and the transitions of *m/z* 399 to 80 or 169 were monitored, 1∼3 chromatographic peaks before the linear isomer (*n*-) PFHxS were observed and suggested as branched isomers [22–25]. In this study, as methanol was used as an organic solvent in the mobile phase B, the TIC (Figure 1(a)) of *Br*-PFHxSK was similar to a previous study [25], exhibiting four peaks (M1-M4) from the latest to earliest elution order. However, as acetonitrile was used in mobile phase B, five peaks (A1-5) were resolved (Figure 1(b)). Moreover, the separation between A2 and A3 (Resolution of chromatographic peaks, *R* = 1.1) was improved relative to that of M2 and M3 (*R* = 0.98).

The results of ^19^F-NMR indicated that the ECF PFHxS product generally contained four monomethyl-substituted isomers (*1m*-, *2m*-, *3m*-, and *iso*- (*4m*-)) and a couple of di-substituted isomers such as *3,3m*-PFHxS [33]. By using the authentic linear standard, A1 at 8.03 min (or M1 at 7.27 min) in the TIC was easily identified as *n*-PFHxS, which showed the strongest product ion *m/z* 80, corresponding to SO_3_^−^ (Figure S1). As PFOS was separated on the same column, the elution order was di-substituted- < 3*m*- < 4*m*- < 1*m*- ≈ 2*m*- ≈ *5m*- < *iso*- < *n*-PFOS [13, 32, 34]. If PFOS and PFHxS have similar elution behaviors, A2-5 (M2-4) were deemed to be the branched isomers of PFHxS. Among them, the earlier eluting peaks could correspond to the di-substituted isomers, while the latter ones corresponded to the mono-substituted isomers, and *iso*-PFHxS should be in the peak of A2 or M2 because *iso*-PFHxS should be the closest to *n*-PFHxS.

**Figure 2:**
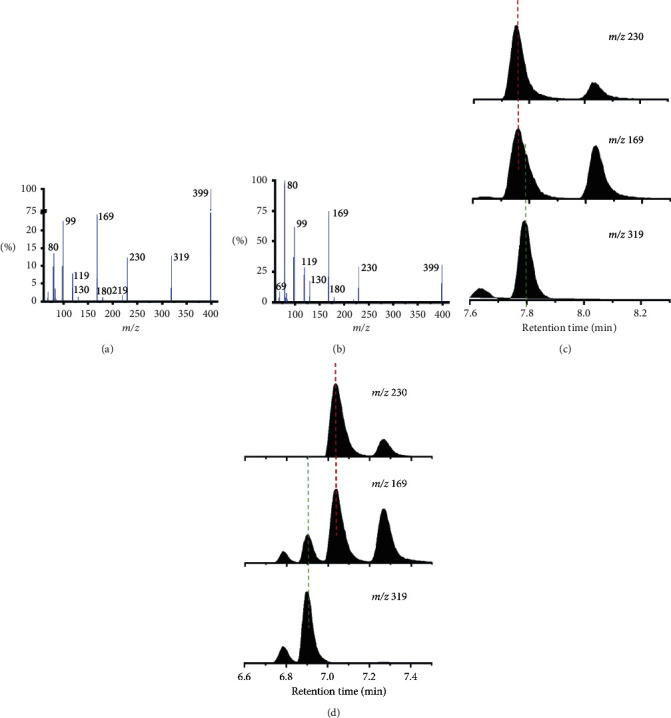
Mass spectra of chromatographic peaks A2 (a) and M2 (b) presented in Figure 1; Chromatographs of typical fragment ions of peak A2 (c), and peak M2 and M3 (d) reflecting the compositions of two PFHxS isomers.

In the MS/MS spectra, A2 (about 7.77 min) generated a series of product ions at *m/z* 169, 230, and 319 (Figure 2(a)), but M2 (about 7.03 min) was characterized by highly abundant fragment ions at *m/z* 169 and 230 without *m/z* 319, while the *m/z* 319 ion was co-eluted earlier in M3 at 6.90 min (Figure 2(b) and Table 2). Therefore, the A2 peak might contain two isomers, one of them has two specific product ions at *m/z* 169 and 230, while another is characterized by the abundant *m/z* 319 ion.

**Scheme 1:**
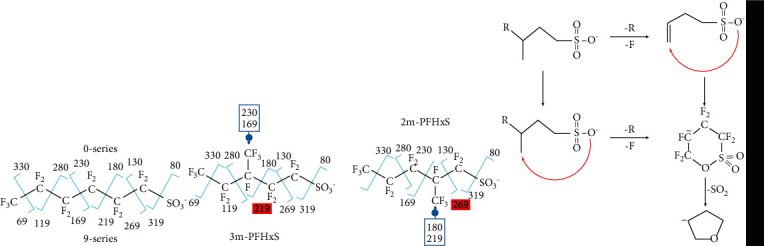
(a). Fragmentation patterns of the production ions generated from PFHxS molecular ions (*m/z* 399); (b). Fragmentation patterns of the molecular ions of 3*m*- and 2*m*-PFHxS: The arrow marks the missing product ion and the enhanced production ions corresponding to the preferential cleavage near the position of CF_3_ substitution; (c) proposed mechanism of 1-series product ion at *m/z* 261.

According to existing studies on the fragmentation rule of PFOS, “9-series” and “0 series” can be used as qualitative ions for isomer identification (Scheme 1) [35]. Stability of the charged ions produced by the secondary or ternary carbonium ions from the mono- or di-substituted C-atom can result in enrichment of the specific 9-series. For example, 1*m*-PFOS could be identified unequivocally due to the presence of highly abundant fragment *m/z* 419, produced by the inductive cleavage of the bond between the SO_3_^−^ and adjacent C-atom. Following the same rule, 1*m*- and *iso*-PFHxS should produce prominent fragments at *m/z* 319 and 169, respectively. Moreover, due to the CF_3_- substitution in branched chains, a certain “9-series” and “0-series” should be missing. Thus, the positions of the branched chains can be determined by inspecting all the enhanced product ions and missing product ions due to the specific substitution, see Table S3. Combining the elution order of all isomers and the characteristic fragments, one of the two isomers eluted in peak A2 was tentatively assigned as *iso*-, which contains a strong product ion at *m/z* 169 but without *m/z* 319, the other isomer in peak A2 was suggested as 1*m*-PFHxS, which has a strong product ion *m/z* 319.

M4 contained several product ions of 9-series (Figure S2A), while A4 was characterized by highly abundant fragment ions at *m/z* 119, 169, and 319 but without *m/z* 219 and 269. A5 was characterized by highly abundant fragment ions at *m/z* 219 and extremely weak or missing at *m/z* 119, 169, and 319 (Figure S2B). Comparing the MS/MS spectra of M4, A4, and A5, M4 seems to be a composite of A4 and A5. To verify this speculation, the elution components of M4 were collected and concentrated, and then separated with the mobile phase containing acetonitrile. Two peaks corresponding to A4 and A5 at 7.53 min and 7.36 min, respectively, were observed. Due to the earliest elution among all the isomers, M4 (A4 and A5) were deemed as two di-substituted isomers. According to the substitution effect on the fragmentation pattern listed in Table S3, the di-substituted isomer 3,3*m*-PFHxS should generate distinct *m/z* 219 ion without *m/z* 119 and 169 ions. On the other hand, the distinction of *m/z* 319 ion with missing of *m/z* 219 and 269 ions should be the feature of *1,1m*-PFHxS. Therefore, A4 and A5 were assigned as *1,1m*- and *3,3m*-PFHxS, respectively.

Through the above analyses, the two isomers (*2m*- and *3m*-) were not yet identified. According to Scheme 1(b), *3m*-PFHxS might display an enhanced signal at *m/z* 219 but without *m/z* 169, and the enhanced signal at *m/z* 269 without *m/z* 219 matches the feature of *2m*-PFHxS. Besides 0-series and 9-series, 1-series fragments such as *m/z* 261 also serve as an important hint for isomer appointment. Langlois and Oehme observed the product ion at *m/z* 261 in the spectrum of 3*m*-PFOS and they speculated that it might be formed by a 1,4-elimination, resulting in a charged alkenesulfate (I, Scheme 1(c)) [36]. However, they did not observe this phenomenon for other isomers. According to Lyon et al. and Cooper et al. [37, 38], the ion with *m/z* 261 might not be the unstable alkenesulfate (I), but the more stable 6-membered ring species produced by the subsequent nucleophilic substitution, which could further transform to the fragment with *m/z* 197 by loss of SO_2_. Another alternative mechanism for the formation of *m/z* 261 might be a result of nucleophilic attack of the negative terminal group at the primary C-atom in CF_3_- linked to the branch of the molecular ion with *m/z* 399, accompanied by the synchronous loss of F^−^ and fluorocarbon-chain *R* [21, 32]. Following this mechanism, for 2*m*-PFHxS, a five-membered fragment ion with *m/z* 211 and its subsequent ion with *m/z* 147 should be formed.

**Figure 3:**
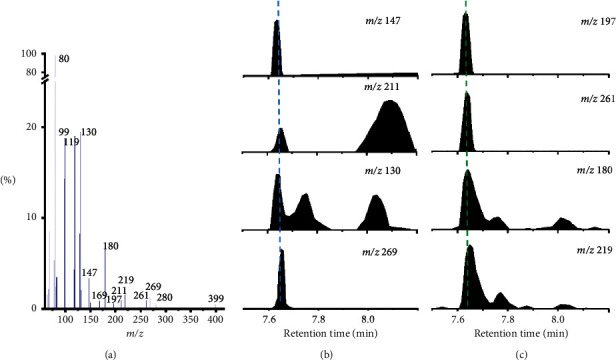
Mass spectra of chromatographic peak A3 (a) presented in Figure 1; Chromatographs of typical fragment ions of peak A3 (B and C), reflecting the compositions of two PFHxS isomers.

Referring to the elution order of PFOS isomers, the 2*m-* and 3*m*- PFHxS isomers should be eluted as A3 peak between the *iso-, 1m-,* and di-substituted isomers we have identified [32]. The mass spectrum of A3 presented multiple fragment ions with *m/z* 319, 269, 261, 219, 211, 197, 180, 169, 147, 130, 99, 80, etc (Figure 3(a)). The abovementioned fragment ions of peak A3 were thus divided into two groups: Group I (*m/z* 269, 130, 211, and 147) (Figure 3(b)), Group II (*m/z* 219, 180, 261, and 197) (Figure 3(c)) based on the 2*m-* and 3*m-*PFOS fragment appointment [32], which were tentatively appointed to *2m*- and *3m*-PFHxS, respectively (Table 2). The characteristic ions *m/z* 180 and 261 in Group II can be exclusively assigned to *3m*-PFHxS, but not *2m*-PFHxS [19]. Theoretically, *2m*-PFHxS should produce special fragments at *m/z* 169, 230, 211,147, and 169, but without the ions *m/z* 180 and 261. However, *2m*-PFHxS was not only the relatively low content (about 1%), but also the co-elution with 3*m*-PFHxS, which resulted in its characteristic ions being difficult to identify. The situation of 2*m*-PFHxS was just the same as that of 2*m*-PFOS, in which appointment and quantification were usually neglected for isomer profiling [19, 36].

### 3.2. Comparison between NMR and LC-MS/MS Quantitative Methods

According to the above appointment of characteristic ions of PFHxS isomers, these fragment ions with strong abundance, i.e., *m/z* 80 at 8.05 min, *m/z* 169 at 7.05 min, *m/z* 180 at 7.63 min, and *m/z* 319 at 6.90 min were exclusively used as quantification ion for *n-, iso-, 3m-,* and *1m-*PFHxS, respectively. For quantification of *2m*-PFHxS, it was recommended to ignore, like *2m*-PFOS. But even to realize its quantification, a new strategy was proposed: to integrate the peak of A3 by *m/z* 80 at 7.60 min as the sum of 2 + 3*m-*PFHxS, and then the content of 2*m-*PFHxS was estimated by deducting the amount of *3m-*PFHxS from the sum, which was carefully verified by NMR quantitative comparison.

**Figure 4:**
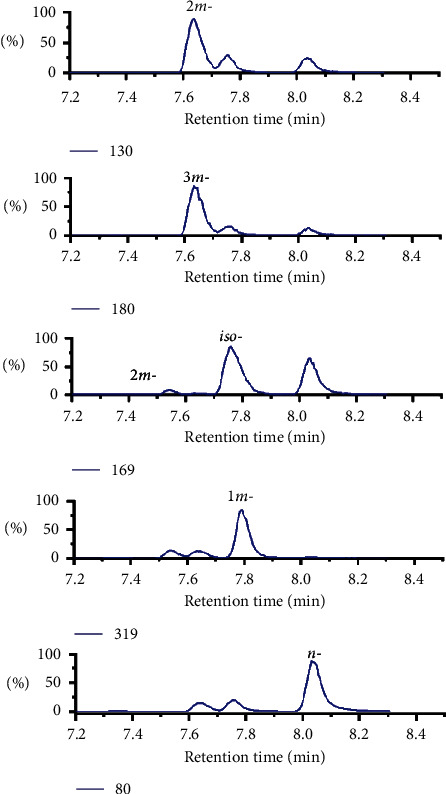
Identification of the isomers in the standard based on the observed specific series of product ions produced from the molecular ion *m/z* 399 under MRM mode.

Figure 4 illustrates the specific MRM daughter ions to quantify the individual isomers.

The optimized MS/MS conditions are listed in Table 3. Under these conditions, the elution order of monomethyl-substituted PFHxS isomers in acetonitrile phase followed the trend of 2*m-* *≈* 3*m-* *<* *iso-* *≈* 1*m-* *<* *n*-PFHxS, which was generally consistent with those of PFOS isomers [32].

**Figure 5:**
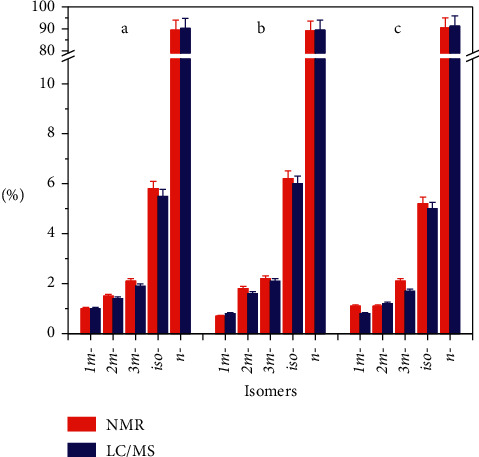
Comparison of the isomer proportions of PFHxS in three technical products (a Bidepharm; b CNW; c J&K Scientific) measured by NMR and LC/MS independently.

A series of *br-*PFHxK solutions with an internal standard of ^18^O_2_-*n*-PFHxS were prepared and the calibration curves for all the isomers were obtained with *R* > 0.99 (Table 4). The three technical products were selected as verification references due to their high purity (>98%, provided by the supplier) (Table S2). Their isomer compositions were determined by ^19^F-NMR and the established LC-MS/MS method, and the results are compared in Figure 5. The established LC-MS/MS method generated every consistent result for the isomers determined by ^19^F-NMR (*p* < 0.05, *t*-test), magnifying that the structural identification was correct, and the quantitation was satisfactory.

### 3.3. Environmental Application

**Figure 6:**
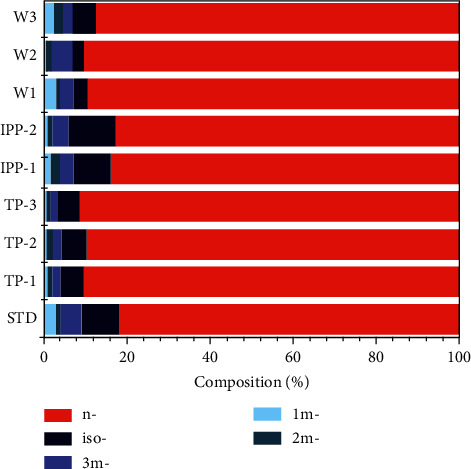
Comparison of compositions of PFHxS isomers in a standard (STD) from Wellington, three PFHxS technical products (TP-1, Bidepharm; TP-2, CNW; TP-3, J&K Scientific), two PFOS industrial products containing PFHxS as impurity (IPP-1, Wuhan Haide Co. Ltd. (Wuhan, China); IPP-2, Qinhuangdao Bainaite Technology (Qinhuangdao, China) and Guangdong Rongxiang Technology (Dongguan, China)) and three water samples from different sources (W1, Haihe Rivers (*n* = 3); W2, Taihu Lake (*n* = 2); W3, drinking water from Tianjin city, China).

Figure 6 illustrates the measured data on the isomeric compositions of PFHxS in the three PFHxS technical products, two PFOS industrial products, and seven environmental water samples. The mean PFHxS isomer profiles in the three PFHxS technical products were 90.4% (*n*-), 5.5% (*iso*-), 1.9% (3*m*-), 1.3% (2*m*-), and 0.9% (1*m*-). In the PFOS commercial industrial products which contains 10–17% of PFHxS as impurity [6], the isomeric pattern of PFHxS was 83.2% (*n*-), 10.1% (*iso*-), 3.5% (3*m*-), 2.0% (2*m*-), and 1.3% (1*m*-). Apparently, the isomeric compositions of PFHxS as an impurity in PFOS industrial products were distinctly different from those of PFHxS own industrial product.

The established LC-MS/MS method was then applied to measure PFHxS isomers in the environmental water samples. Several typical surface waters collected from Haihe River and Taihu Lake, China, where the occurrence of PFAAs including total PFHxS was reported previously [30, 31]. Two local tap water samples were also collected for isomer profiling. Their concentrations are shown in Table 5 and were between 1 and 5 *ng*/L. The method detection limits (MDLs) for all isomers in water were in the range of 4 to 30 *pg*/L (Table 4), which were similar to those of *n*-PFOS reported in previous studies (1–3 *pg*/L) [40–42]. Among these water samples, except that 1*m*-PFHxS was not identified in some samples, all other isomers were detected. In the surface waters and drinking waters, the mean isomer profiles of PFHxS included 90.0% of *n*-, followed by 4.4% of *iso*-, 3.0% of 3*m*-, 1.5% of 2*m*-, and 2.0% of 1*m*-PFHxS. The isomeric compositions of PFHxS in the waters were more alike to the PFHxS technical products rather than that impurity in the PFOS industrial products (Table S4). These results implied that the discharge from direct usage of PFHxS product, which is being increasingly used as PFOS substitute, maybe a predominant contribution to PFHxS in the environment. This supports the result of a previous study, which investigated the source of PFHxS in the Yellow Sea coastal water, China [30].

## 4. Conclusions

The established method is useful for measuring trace amounts of PFHxS isomers, for the sake of source tracking and evaluating possible differences in toxicology and environmental behaviors among PFHxS isomers. Without derivatization, the method can be applied to routine environmental sample analyses. The isomer profiling of PFHxS not only provides sufficient information on the sources of PFHxS in the environment, but also supplies evidence of the isomer-selective behaviors and effects of PFHxS.

## Figures and Tables

**Table 1:** Gradient eluting procedures for isolation of PFHxS isomers.

Time (min)	Flow rate (mL/min)	A (%) aqueous phase(20 mM ammonium formate, pH = 4.0)	B (%) organic phase(methonal or acetonitrile)	Curve
0.0	0.25	90 (90)	10 (10)	6
2.0		90 (90)	10 (10)	6
5.0		40 (70)	60 (30)	6
16.0		12 (30)	88 (70)	6
16.5		8 (0)	92 (100)	6
17.5		0 (0)	100 (100)	6
20.0		90 (90)	10 (10)	6
23.0		90 (90)	10 (10)	6

**Table 2:** Identification of PFHxS isomers based on the observed specific series of product ions produced from the molecular anion *m/z* 399.

Fragment ion series			Main product ions (m/z) observed							
Series	Formula^a^	Possible product ions (*m/z*)	A1b	A2	M2	A3		M3	A4	A5
			M1b									M4
9-Series	[C_n_F_2n+1_]^−^	69, 119, 169, 219, 269, 319		169, 319	169^*∗*^	169	219	319^*∗*^	319 [219, 269]c	219 [119, 169]^c^

0-Series	[C_n_F_2n_SO_3_]^−^	80, 130, 180, 230, 280, 330, 380	80^*∗*^	230	230	130, 80^*∗*^	180^*∗*^			

1-Series	[C_n_F_2n−1_ SO_3_]^−^	111, 161, 211, 261				211	261	161		

7-Series	[C_n_F_2n−1_O]^−^	47, 97, 147, 197				147	197			

PFHxS isomer			*n-*		iso-	2m-	*3m-*	1m-	1, 1m-	3, 3m-

^a^: *n* = 1, 2,…, 6. ^b^: Identified by comparison with the commercially available *n-*PFHxS standard and its quantitative ion was *m/z* 80. ^**c**^: Fragment ions that cannot be produced due to the position of the branched chain. ^*∗*^: The specific product ions were chosen to quantify the corresponding isomers.

**Table 3:** Conditions of the MS/MS for isolation of target analytes and corresponding internal standard for quantification.

Chemicals	Transitions	Cone voltage (V)	Collisionvoltage (V)	Internal standard	Transitions	Conevoltage (V)	Collisionvoltage (V)
n-PFHxS	399 ⟶ 80^*∗*^	40	30	^18^O_2_-n-PFHxS	403 ⟶ 84^*∗*^	40	30
1m-PFHxS	399 ⟶ 161		25				
	399 ⟶ 319^*∗*^		25				

2m-PFHxS	399 ⟶ 80^*∗*^		30				
	399 ⟶ 211		25				

3m-PFHxS	399 ⟶ 261		35				
	399 ⟶ 219		35				
	399 ⟶ 180^*∗*^		25				

4m-PFHxS	399 ⟶ 169^*∗*^		35				
	399 ⟶ 230		25				

^*∗*^: for quantification.

**Table 4:** Quality control parameters for PFHxA isomers.

Compound	Isomer	Linear range (*µ*g/L)^a^	IDL (ng/L)	Linear range (*µ*g/L)^b^	MDL (ng/L)	Recovery (%)
PFHxS	*n-*	0.81–162.2	0.01	0.041–40.6	0.030	112.37 ± 6.79
	1*m-*	0.029–5.8	0.004	0.015–1.45	0.004	115.86 ± 4.42
	2*m-*	0.014–2.8	0.006	0.007–0.7	0.009	112.79 ± 6.60
	3*m-*	0.05–10.0	0.008	0.025–2.5	0.008	109.83 ± 9.32
	*iso-*	0.089–17.8	0.006	0.045–4.5	0.007	110.91 ± 5.09

^a^: linear detectable ranges of PFHxS isomer were set for measurement of technical products (*R* > 0.9940). ^b^: linear detectable ranges of PFHxS isomer were set for measurement of water samples (*R* > 0.9928).

**Table 5:** Concentration and compositions of water samples.

		1*m*-PFHxS	2*m*-PFHxS	3*m*-PFHxS	*iso*-PFHxS	*n*-PFHxS
Haihe river-1 (J2)^a^	Conc (ng/L)	0.061	0.013	0.028	0.064	1.32
	Proportion (%)	4.1	0.9	1.9	4.3	88.8

Haihe river -2 (Y3)^a^	Conc (ng/L)	0.027	0.017	0.017	0.064	1.05
	Proportion (%)	2.3	1.4	1.5	5.4	89.4

Haihe river -3 (MR1)^a^	Conc (ng/L)	0.072	0.029	0.042	0.13	2.38
	Proportion (%)	2.7	1.1	1.6	4.9	89.7

Taihu lake-1 (W36)^a^	Conc (ng/L)	N.D.	0.079	0.43	0.12	4.63
	Proportion (%)	N.D.	1.5	8.2	2.3	88.1

Taihu lake--2 (W37)^a^	Conc (ng/L)	0.072	0.058	0.067	0.17	4.43
	Proportion (%)	1.5	1.2	1.4	3.6	92.2

Drinking water-1^b^	Conc (ng/L)	0.035	0.043	0.034	0.096	1.39
	Proportion (%)	2.2	2.7	2.1	6.0	87.1

Drinking water -2^b^	Conc (ng/L)	0.035	0.020	0.034	0.058	1.05
	Proportion (%)	2.9	1.7	2.8	4.8	87.8

^a^: The sample points in brackets and treatment procedures, please refer to the literature [30, 31] for details; ^b^: Drinking water collection and treatment methods, see references for details [39]. N.D.: not detected.

## Data Availability

The NMR signal assignment, Nomenclature of main PFHxS isomers (Table S1), Information for the three PFHxS technical products (TP) obtained from China (Table S2), Theoretically missing or enhanced product ions of the “0-series” and “9-series” due to the CF3 substitution for mono-substituted isomers (Table S3), *p* values of isomer compositions of water samples compared with PFHxS technical products and PFHxS impurity of PFOS industrial products using Kruskal–Wallis test (Table S4), Mass spectra (A) of A1 and the chromatograms (B) of technical PFHxS product and n-PFHxS standard solution, confirming the peak at retention time at 8.05 min was n- PFHxS (Figure S1), Mass spectra of chromatographic peak A4, A5 and M4, presented in Figure 1 (Figure S2A), Chromatographs of typical fragment ions of peak A5 and peak A4, reflecting the peak position zone of di-substituted isomers(Figure S2B). 19F-NMR of the technical product from Anpel Lab Technologies Inc (Figure S3), data used to support the findings of this study are included within the supplementary information file.

## Supplementary Materials

Supplementary Materials NMR signal assignment: Table S1. Nomenclature of main PFHxS isomers. Table S2. Information for the three PFHxS technical products (TP) was obtained from China. Table S3. Theoretically missing or enhanced product ions of the “0-series” and “9-series” due to the CF_3_ substitution for mono-substituted isomers. Table S4. *p* values of isomer compositions of water samples compared with PFHxS technical products and PFHxS impurity of PFOS industrial products using Kruskal–Wallis test. Figure S1. Mass spectra (A) of A1 and the chromatograms (B) of technical PFHxS product and *n*-PFHxS standard solution, confirming the peak at retention time at 8.05 min was *n*- PFHxS. Figure S2. Mass spectra of chromatographic peaks A4, A5, and M4, presented in Figure 1 (A), Chromatographs of typical fragment ions of peak A5 and peak A4, reflecting the peak position zone of di-substituted isomers (B). Figure S3. ^19^F-NMR of the technical product from Anpel Lab Technologies Inc.
