# Supplementary material for: Analysis of Specific Perfluorohexane Sulfonate Isomers by Liquid Chromatography-Tandem Mass Spectrometry: Method Development and Application in Source Apportionment
Source: J Anal Methods Chem. 2022 Sep 22;2022:8704754. doi: 10.1155/2022/8704754 (PMC9553683; doi:10.1155/2022/8704754)
Supplement: Supplementary Materials — NMR signal assignment: Table S1. Nomenclature of main PFHxS isomers. Table S2. Information for the three PFHxS technical products (TP) was obtained from China. Table S3. Theoretically missing or enhanced product ions of the “0-series” and “9-series” due to the CF3 substitution for mono-substituted isomers. Table S4. p values of isomer compositions of water samples compared with PFHxS technical products and PFHxS impurity of PFOS industrial products using Kruskal–Wallis test. Figure S1. Mass spectra (A) of A1 and the chromatograms (B) of technical PFHxS product and n-PFHxS standard solution, confirming the peak at retention time at 8.05 min was n- PFHxS. Figure S2. Mass spectra of chromatographic peaks A4, A5, and M4, presented in Figure 1 (A), Chromatographs of typical fragment ions of peak A5 and peak A4, reflecting the peak position zone of di-substituted isomers (B). Figure S3. 19F-NMR of the technical product from Anpel Lab Technologies Inc. [file 8704754.f1.docx]

**Supporting Information for**

**Analysis of Specific Perfluorohexane Sulfonate Isomers by Liquid Chromatography-Tandem Mass Spectrometry: Method Development and Application in Source Apportionment**

Liping Yang, Xin Chen, Lingyan Zhu, Yixin Wang, Guoqiang Shan^^[[1]](#footnote-1)^*^

Key Laboratory of Pollution Processes and Environmental Criteria, Ministry of Education, Tianjin Key Laboratory of Environmental Remediation and Pollution Control, College of Environmental Science and Engineering, Nankai University, Tianjin, P. R. China 300350

**NMR signal assignment**

Chemical shifts were reported in ppm relative to hexafluorobenzene using the signal at -169 ppm according to previous study ^[1]^. All ^19^F-NMR analysis were repeated in triplicate. The purity and proportions of mono-substituted isomers of PFHxS were determined using hexafluorobenzene as internal reference.

The NMR spectra of technical products of PFHxS are shown in Fig S6. According to the previous method ^[1]^, ^19^F-NMR analysis of technical PFHxS was carried out by integration of specific isolated signals [***n*-**: C-6 at -86.2 ppm (after abstraction of *2m*-, *3m*-); ***1m***-: C-1 at -174.6 ppm; ***2m*-**: branched CF_3_ at -74.4 ppm; C-1 at -108.7 and -109.2 ppm; C-2 at -187.0 ppm; ***3m*-**:branched CF_3_ at -75.5 ppm; C-1 at -116.3 ppm C-6 at -130.6 ppm; ***iso*-:** C-1 at -119.3 ppm, branched CF_3_ at -76.9 ppm; *t*-butyl group at -66.6 ppm.

**Table S1. Nomenclature of main PFHxS isomers**

**Table S2.** Information for the three PFHxS technical products (TP) obtained from China

| Technical products | Company | PFHxS content (%) |
| --- | --- | --- |
| TP-1 | Bidepharm Inc. | ≥98 |
| TP-2 | Anpel lab technologies Inc. | ≥98 |
| TP-3 | J&K Scientific Co. Ltd. | ≥98 |
| IPP-1 | Qinhuangdao Bainaite Technology Co. | 15.9^a^ |
| IPP-2 | Guangdong Rongxiang Technology | 17.1^a^ |

^a^: see the literature^[2]^.

**Table S3:** Theoretically missing or enhanced product ions of the “0-series” and “9-series” due to the CF_3_ substitution for mono-substituted isomers

| Isomer | Missing product  ion of 0-series | Enhanced product  ion of 9-series | Missing product  ion of 9-series |
| --- | --- | --- | --- |
| *1m*-PFOS | 130 | 419 |  |
| *2m*-PFOS | 180 | 369 |  |
| *3m*-PFOS | 230 | 319 |  |
| *4*m-PFOS | 280 | 269 |  |
| *5*m-PFOS | 330 | 219 |  |
| *6m*(*iso*)-PFOS | 380 | 169 |  |
| *1m*-PFHxS | 130 | 319 |  |
| *2m*-PFHxS | 180 | 269 | 219 |
| *3m*-PFHxS | 230 | 219 | 169 |
| *4m*(*iso*)-PFHxS | 280 | 169 |  |
| *3,3m*-PFHxS |  | 219 | 119,169 |
| *1,1m*-PFHxS |  | 319 | 219,269 |

**Table S4.** *p* values of isomer compositions of water samples compared with PFHxS technical products and PFHxS impurity of PFOS industrial products using Kruskal-Wallis test.

|  | Isomer | PFHxS technical product | PFHxS Impurity of PFOS industrial products |
| --- | --- | --- | --- |
| Water samples | *1m*-PFHxS | 0.05* | 0.01** |
|  | *2m*-PFHxS | 1.00 | 0.11 |
|  | *3m*-PFHxS | 0.83 | 0.30 |
|  | *iso*-PFHxS | 0.06 | 0.02* |
|  | *n*-PFHxS | 0.11 | 0.02* |

* represents *p* < 0.05

** represents *p* < 0.01


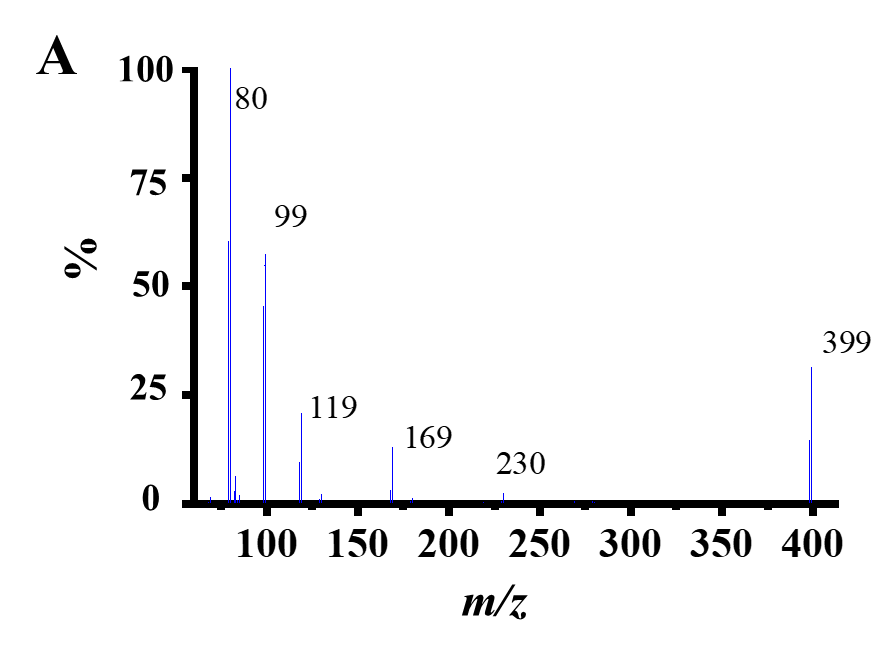


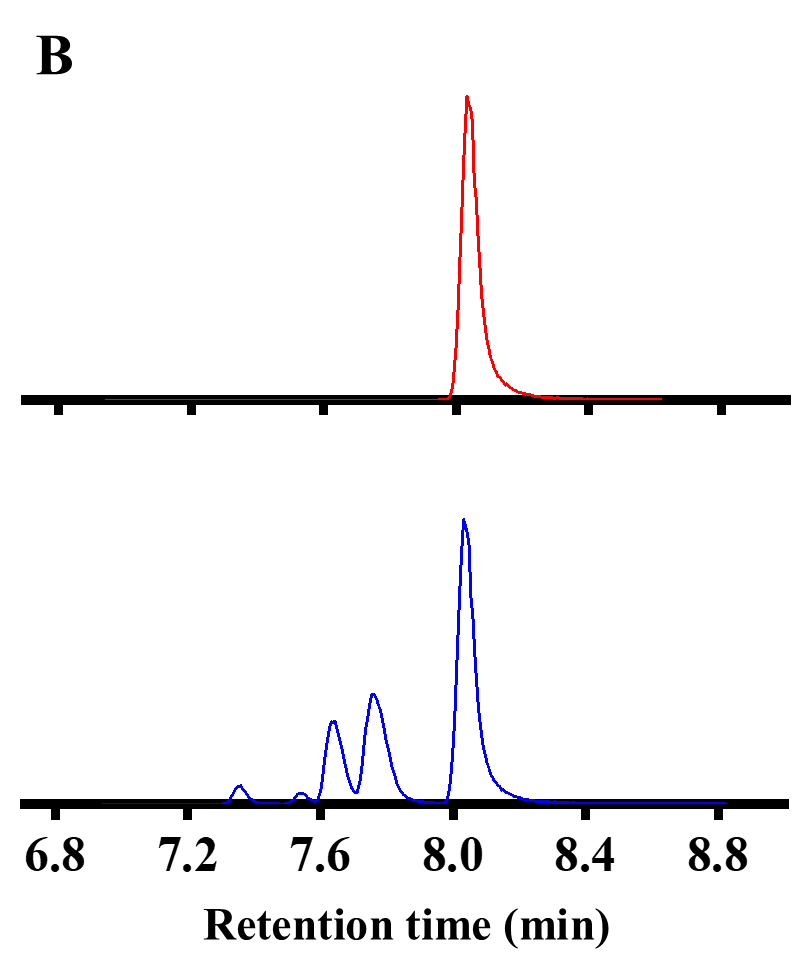


**Fig S1**. Mass spectra (A) of A1 and the chromatograms (B) of technical PFHxS product and *n*-PFHxS standard solution, confirming the peak at retention time at 8.05 min was *n*- PFHxS.


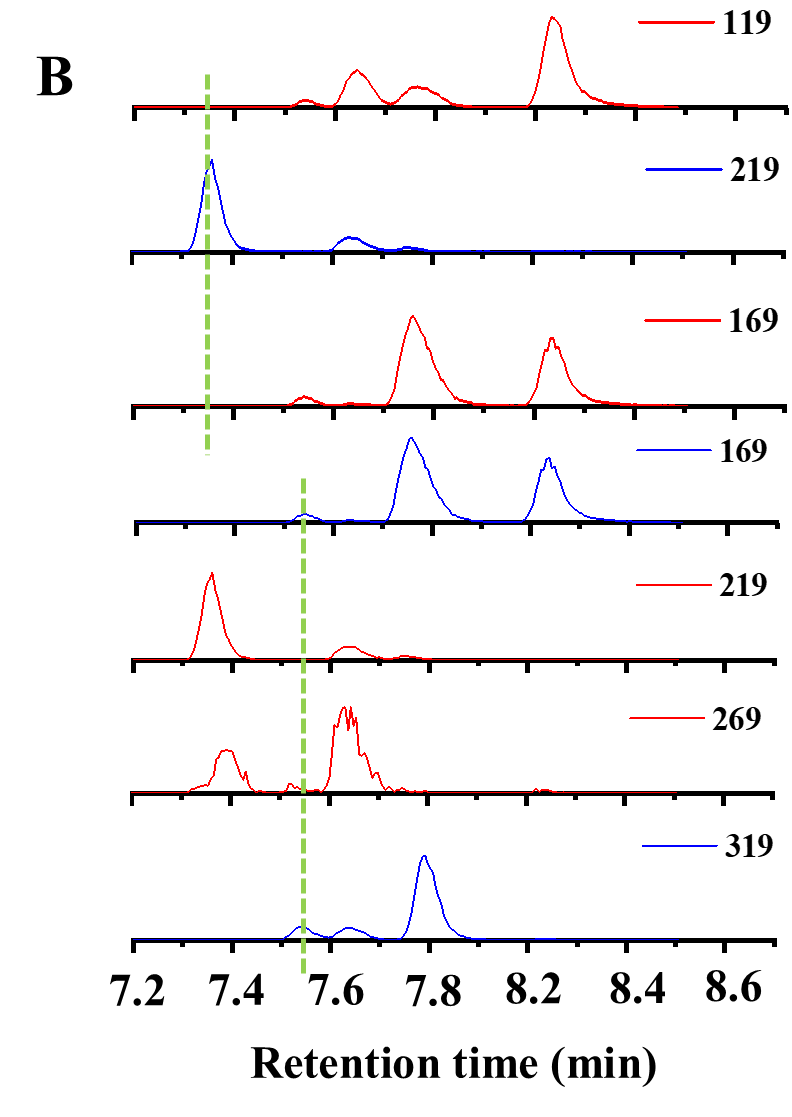


**Fig. S2.** (A) Mass spectra of chromatographic peak A4, A5 and M4, presented in Fig. 1; (B); Chromatographs of typical fragment ions of peak A5 and peak A4, reflecting the peak position zone of di-substituted isomers.

**Fig. S3. ^19^F-NMR of the technical product from Anpel Lab Technologies Inc.**

1. CF_3_ region
2. CF_2_ region
3. CF region

References:

1. Arsenault G,Chittim B,Gu J,McAlees A,McCrindle R.Robertson V. Separation and fluorine nuclear magnetic resonance spectroscopic (19F NMR) analysis of individual branched isomers present in technical perfluorooctanesulfonic acid (PFOS). Chemosphere, 2008, 73(1 Suppl): S53-9

2. Jiang W,Zhang Y,Yang L,Chu X.Zhu L. Perfluoroalkyl acids (PFAAs) with isomer analysis in the commercial PFOS and PFOA products in China. Chemosphere, 2015, 127: 180-7

3. Feng X,Ye M,Li Y,Zhou J,Sun B,Zhu Y.Zhu L. Potential sources and sediment-pore water partitioning behaviors of emerging per/polyfluoroalkyl substances in the South Yellow Sea. J Hazard Mater, 2020, 389: 122124

4. Li Y,Feng X,Zhou J.Zhu L. Occurrence and source apportionment of novel and legacy poly/perfluoroalkyl substances in Hai River basin in China using receptor models and isomeric fingerprints. Water Res, 2020, 168: 115145

5. xin C. Contents of Li, Se, Mo and Cr in Bt corns. Spectrosc Spect Anal, 2021, 27(8): 1638-1639

1. * Corresponding author, E-mail address: [yunongshan@nankai.edu.cn](mailto:yunongshan@nankai.edu.cn); Phone: +86-22-23500791. Fax: +86-22-23503722. [↑](#footnote-ref-1)
